# Low-Power Detection of Food Preservatives by a Novel Nanowire-Based Sensor Array

**DOI:** 10.3390/foods8060226

**Published:** 2019-06-25

**Authors:** Dario Zappa

**Affiliations:** SENSOR Laboratory, DII, Università degli Studi di Brescia, Via Valotti 9, 25133 Brescia, Italy; dario.zappa@unibs.it; Tel.: +39-030-371-5767

**Keywords:** metal-oxides nanowires, chemical sensing, sensor array, electronic noses, food preservation

## Abstract

Food preservatives are compounds that are used for the treatment of food to improve the shelf life. In the food industry, it is necessary to monitor all processes for both safety and quality of the product. An electronic nose (or e-nose) is a biomimetic olfactory system that could find numerous industrial applications, including food quality control. Commercial electronic noses are based on sensor arrays composed by a combination of different sensors, which include conductometric metal oxide devices. Metal oxide nanowires are considered among the most promising materials for the fabrication of novel sensing devices, which can enhance the overall performances of e-noses in food applications. The present work reports the fabrication of a novel sensor array based on SnO_2_, CuO, and WO_3_ nanowires deposited on top of μHPs provided by ams Sensor Solutions Germany GmbH. The array was tested for the discrimination of four typical compounds added to food products or used for their treatment to increase the shelf life: ethanol, acetone, nitrogen dioxide, and ozone. Results are very promising; the sensors array was able to operate for a long time, consuming less than 50 mW for each single sensor, and principal component analysis (PCA) confirmed that the device was able to discriminate between different compounds.

## 1. Introduction

Nowadays, we are living in an era of images in which vision is considered the most important of the human senses. Nevertheless, vision is only one of the five human senses: vision, hearing, olfaction, taste, and touch. Among these senses, olfaction is certainly the most mysterious and complex one, even if it has historically been considered of a lower status in relation to the other senses [1]. Apparently, olfaction in humans has lost the importance that it retains in animals, as it is often involved during prey hunting and feeding [2]. However, it is one of our innate warning systems in the case of danger, for example, in the case of fire. To give an idea of the importance of the olfactory systems, almost 4% of the genomes of many higher eukaryotes are devoted to encoding the proteins that are responsible for smell [3]. Human beings possess an excellent ability to detect and discriminate odors, but they typically have great difficulty in identifying particular odorants. 

Artificial olfaction (also called electronic nose or e-nose) is a biomimetic olfactory system [4,5,6] that could find numerous industrial applications, such as indoor air and environmental monitoring [7,8,9], hazardous gas detection and customs security [10,11], medical care [12,13,14], and food quality control [15,16,17]. It is possible to identify at least two main areas in the food industry that benefit from the use of an artificial olfaction system. The first one is the detection of contaminants during food processing chains, as the preemptive assessment of the source of contaminants is not always possible [18,19,20]. Contaminating microorganisms may enter and reach the end-product through many parallel routes, such as though raw materials, air in the processing plant area, process surfaces, or even inadvertent personnel. The second application of e-noses in the food industry is the quality control of raw materials and end-products, and, in particular, authenticity [21,22,23], food freshness [24], the presence of allergens, and shelf life [25]. The increased demand for long-term storage and preservation creates the need to develop methods that can easily track and assess food freshness. Moreover, authenticity of food is a rising issue in many countries, even market leaders. As an example, it is estimated that the rate of fraud of Parmigiano Reggiano cheese, a well-known Italian product, is between 20% and 40% worldwide, reaching a dramatic 95% in the United States [23,26].

Commercial electronic noses are based on sensor arrays composed by a combination of different sensors, which include conductometric [27,28], piezoelectric [29,30], field effect transistors [31], optical sensors [32] and many more [33], based on different materials and working principles. Among these, metal oxide (MOX) materials represent the current state of the art in the chemical sensing technology. In 1991, Yamazoe demonstrated that reducing the size of metal oxide materials to nanoscale could lead to a substantial enhancement of their sensing properties [34]. Among these novel nanostructured materials, nanowires are considered among the most promising due to their extremely high surface-to-volume ratio and their unique electrical and chemical properties [35,36,37,38]. 

As with all other materials, metal oxides have some limitations that inhibit an even larger diffusion as sensitive materials [39]. One of the critical requirements is thermal activation—chemical reactions that take place on their surface are promoted by temperature (usually 200–400 °C). Therefore, a substantial part of the energy consumption of an electronic nose is related to its sensors heating. Thanks to the advances in the silicon industry, there are now different micro-machined (MEMS) silicon micro hotplates (μHPs) available on the market, which consist of a thin membrane integrating both interdigited electrodes and a heating element [40]. These substrates allow a drastic reduction of the energy consumption, moving from hundreds of mW to a few tens of mW during continuous operation. Moreover, these hotplates exhibit a very small thermal inertia, which enables the possibility of using smarter sampling techniques and further power saving operating modes, such as fast temperature modulation and discontinuous operation [41,42,43]. 

The aim of the present work is to demonstrate the effective detection of different food preservatives, which are compounds that are used for the treatment of food to improve the shelf life. For this reason, a novel low-power sensor array was fabricated, which is intended as the heart of a more efficient electronic nose instrument. The proposed array integrates three different types of metal oxide nanowires (SnO_2_, CuO, and WO_3_) on top of μHPs provided by ams Sensor Solutions Germany GmbH (72770 Reutlingen, Germany). These nanowires were directly synthetized on top of the hotplates by different techniques, demonstrating the compatibility of the process with mass production. The morphology and the structure of the sensing materials were investigated briefly, and the ability of the array to discriminate among different food preservatives (ethanol, acetone, nitrogen dioxide, and ozone) was tested. 

Ethanol is a compound largely found in the food industry, such as in food fermentation processes and in alcoholic beverage production [44]. However, it is often used as a preservative to increase shelf life as well, as the deliberate addition of low concentrations of ethanol inhibits the proliferation of many microorganisms, increasing the shelf life of packaged food [45,46]. For example, the addition of ethanol at levels between 0.5–3.5% to a loaf of bread leads to a substantial extension of the shelf life of the bread (more than 1000%) [47]. 

Acetone, however, is traditionally used as a solvent in many industrial processes, including acetone-butanol-ethanol (ABE) fermentation of soluble and hydrolyzed sugars [48], and is one ketone that is present as an aroma compound in many foods [49,50]. However, it is a well-known interfering compound in the detection of ethanol, as it is present in many food processes and is quite different to discriminate from ethanol at low concentrations by artificial olfaction systems.

Nitrogen dioxide is a hazardous compound that has to be monitored in many environmental applications. In the food industry, it is the main source of nitrite, which is a very popular and diffuse compound, for example, in aquaculture [51]. In small quantities, it is often added to alcoholic beverages and perishable food such as salami, ham, and meat [52]. In fact, it is a strong antimicrobial agent, but it can have noxious effects on the human body at concentrations higher than safety standards, binding with hemoglobin and therefore reducing the capability of blood to transport oxygen [53,54]. For drinking water, the US Environmental Protection Agency and the World Health Organization set a standard of about 3 mg nitrite/L for short-term exposures [53].

Finally, ozone is a compound that attracted a significant interest in the last 30 years due to its role in the atmosphere. However, ozone is used in many industrial fields, including as an alternative to conventional fermentation processes [55] and as a powerful antimicrobial agent [56]. The application of low amounts of ozone (5–10 mg/L) has been tested as an intervention for eliminating pathogens (*Salmonella, Escherichia coli*) from the surface of different seeds and sprouts [57,58] and as disinfectants of fruit juices [59] and fresh carrots [60], for example, without leaving residues on food products.

## 2. Materials and Methods

**Figure 1:**
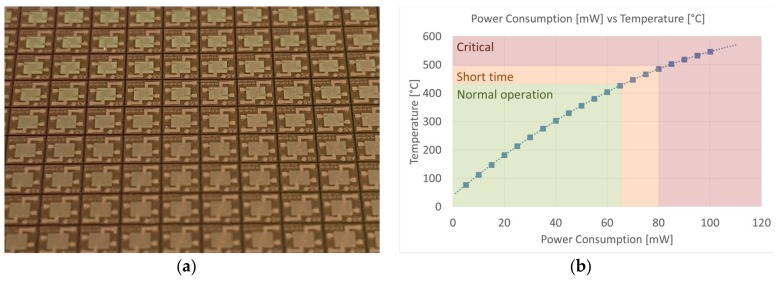
(**a**) Optical image of a wafer of ams E120:20 μHPs. (**b**) Power consumption versus temperature calibration.

Commercial state-of-the art μHPs were provided by ams Sensor Solutions Germany GmbH, a leading semiconductor company that recently entered the market of chemical sensors. Micro hotplates were fabricated using planar silicon technology, allowing the preparation of wafers (thickness 450 µm) of pre-etched substrates that had to be diced if required (Figure 1a). Each micro hotplate was 2 mm × 2 mm size, while the membrane area was 1 mm x 1 mm with a thickness of only 1 μm. Electrodes were deposited on top of the silicon nitride membrane in E120:20 configuration (120 μm × 20 μm), and the heating element was integrated in the membrane itself. According to the specs, these μHPs could work at up to 450 °C for long-term normal operation and up to 500 °C for short bursts if the integrated heating element is used. However, the hotplates were stressed at a much higher temperature (up to 870 °C) inside the furnace during the synthesis of the nanostructures, and they were able to sustain it without any critical breakdown. This was an excellent result considering the very low thickness and the apparent fragility of the membranes. Figure 1b reports the power versus temperature curve of the integrated heater. To reach a temperature of 400 °C, only 60 mW were required. As a comparison, sensors prepared by using 2 mm × 2 mm alumina substrates that are integrated in commercial electronic noses consume ten times more energy [61]. 

### 2.1. Synthesis of Tungsten Trioxide (WO_3_) Nanowires

Thermal oxidation techniques were used to synthesize tungsten oxide (WO_3_) nanowires on ams E120:20 micro hotplates (μHPs). The synthesis process mainly consisted of three steps—deposition of the metal layer, oxidation, and thermal annealing [62]. 

A thin layer (100 nm) of metallic tungsten was deposited on the hotplates by radio frequency (RF) magnetron sputtering (100 W argon plasma, 5.5 × 10^−3^ mbar, room temperature). Afterwards, samples were oxidized in a tubular furnace (custom design based on Lenton furnaces, UK) at 600 °C for one hour in order to grow the nanowires. The pressure inside the alumina tube was set at 0.8 mbar, inletting an argon flow of 10 SCCM by using MKS (Germany) massflow controllers.

After the growing process, samples underwent a thermal annealing to completely oxidize the material [62]. Samples were heated at 400 °C for 12 h in air at atmospheric pressure in the same tubular furnace. 

### 2.2. Synthesis of Copper Oxide (CuO) Nanowires

The same thermal oxidation technique was used to synthesize copper oxide (CuO) nanowires on μHPs [63], but this time, nanowires were synthesized at atmospheric pressure instead of in a vacuum environment. 

Firstly, 500 nm of metallic copper were deposited on the pre-fabricated electrodes by using RF magnetron sputtering (50 W argon plasma, 5.5 × 10^−3^ mbar, room temperature). Afterwards, the samples underwent a forced oxidation in a tubular furnace (Carbolite, UK). Samples were placed in a quartz holder inside the alumina tube at the chosen temperature (300 °C), gas flow, atmosphere composition, and duration. Gas flow was set at 300 SCCM, and oxidation time was fixed at 12 h in order to obtain a dense mat of nanowires on the surface. The atmosphere inside the furnace consisted of a mixture of argon and oxygen (80–20% Ar). The combination of temperature and oxidation time was enough to completely oxidize the material, removing any trace of metallic copper [63]. 

### 2.3. Synthesis of Tin Oxide (SnO_2_) Nanowires

For the synthesis of tin oxide (SnO_2_) nanowires, a custom physical vapor deposition process (PVD) was used. It mainly consisted of an evaporation–condensation process based on the vapor liquid solid (VLS) mechanism [64,65,66,67].

Platinum nanoparticles were deposited on top of the μHPs by direct current (DC) magnetron sputtering (70 W argon plasma, 5.5 × 10^−3^ mbar, room temperature), acting as catalyst seeds for the nucleation of the nanowires. Tin oxide powder (Sigma-Aldrich, 99.9% purity) was dispersed on an alumina holder and put in the middle of the tubular furnace (custom design based on Lenton furnaces, UK), where the temperature was high enough to evaporate the material (1370 °C). Substrates were placed in a colder region of the furnace (870 °C). At this temperature, the catalytic Pt nanoparticles formed droplets that were in liquid form, promoting the condensation of the evaporated material on top of the substrates due to the lower energy required by the process. An argon carrier flow (100 SCCM, MKS massflow) was used to move the cloud of evaporated material towards the substrates. The pressure inside the furnace was kept at 100 mbar, and the deposition time was 5 minutes [68]. 

### 2.4. Morphological and Structural Characterization

The morphology of the prepared nanowires was investigated by using a field emission scanning electron microscope (FE-SEM model LEO 1525, ZEISS) operated at 3–10 keV energy beam. The microscope was coupled with an Oxford energy dispersive x-ray analysis (EDX). Samples were attached with carbon glue to metallic stubs to reduce charging effects due to the electron beam. 

Raman spectra were measured by using a fiber coupled confocal HORIBA optical microscope at 100x magnification. An iHR320 monochromator was configured with a grating of 1800 g/mm and was connected to a Peltier-cooled Synapse CCD. An He-Cd blue laser (442 nm) was focused on the samples to excite the material and promote the Raman scattering. Spectra were recorded in the wavelength range 200–1000 cm^−1^.

### 2.5. Device Fabrication and Functional Characterization

**Figure 2:**
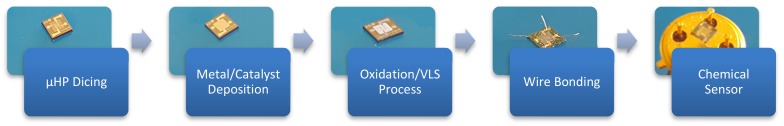
Flow-chart of the integration of metal oxide (MOX) nanowires on ams μHPs to fabricate chemical sensors.

The schematic workflow used for the preparation of the sensing devices is reported in Figure 2. In this experimental work, micro-hotplates were diced individually for greater ease in preparing samples at the prototype level. However, due to the high scalability of the process, all operations could be performed at wafer level, which is a fundamental requirement of mass-scale production of the devices. After the μHPs dicing, a metal/catalyst layer was deposited by magnetron sputtering according to the specific metal oxide nanowires. Finally, samples were oxidized/underwent the VLS process to synthetize the nanowires directly on the hotplates. Functional sensors require a capped hosting case to interface with the external electronics and to protect the sensing element. Therefore, μHPs were mounted on the “transistor outline” TO4 package. Pads were connected to TO pins by electro-soldering 50 µm gold wires.

Different devices of the same batches were prepared, and repeated measurements were performed on nominally identical sensors under the same experimental conditions to evaluate the reproducibility and the yield of the process. The lifetime of the present sensors can be estimated to be >1 year, over which the samples are still working without any evident deterioration of the surface, exhibiting a small drift (<20%) typical of metal oxide materials [69]. It is important to stress that the first cause of device failure is the wrong manipulation—a suspended MEMS membrane is very fragile and could break easily if directly touched. Therefore, samples should be handled with extreme care during wire bonding and during the placing of the protective cap. However, after mounting, sensors are very robust to mechanical stresses, and they are primarily vulnerable only to the thermal shocks if working out of specs, which could result in the breakdown of the membrane or in permanent damage of the electrodes [40,70,71].

To investigate the conductometric response of the sensors, flow-through technique was used. The sensing devices were mounted in a homemade stainless steel test chamber able to measure up to ten sensors simultaneously [72]. The chamber was set at 20 °C to avoid the influence of the external temperature. Humidified air was produced by flowing the dry air through a Drechsel bottle held in a thermostatic bath at 25 °C and then in a condensation vessel in order to favor the condensation of saturated vapor. The humidified air was mixed with dry air in order to obtain the desired relative humidity (RH) content, which was fixed at 50% @ 20 °C (chamber temperature) in these measurements. Sensor temperatures were controlled by modulating the electric power applied to heaters by Thurlbly-Thandar PL330DP power supplies. A 1 V voltage was applied to the sensors, simultaneously measuring the conductance of each sensor using Keithley 6485 picoammetters. 

Prior to measurements, samples were thermally stabilized at the optimal working temperature for each chemical compound for 10 h. Selected gas concentration was let in the chamber for 30 min, followed by a restore with synthetic air flow for 90 min to allow the recovery of the baseline. The response of n-type semiconductor sensors was determined by the variation of the conductance, using, for a reducing gas, the following formula:

(1) $$Response=\frac{R_{Gas}-R_{Air}}{R_{Air}}=\frac{G_{Air}-G_{Gas}}{G_{Gas}},$$

and for an oxidizing gas:

(2) $$Response=\frac{G_{Gas}-G_{Air}}{G_{Air}},$$

where $R_{Gas}$ and $G_{Gas}$ are respectively the resistance and the conductance of the sensor in gas, and $R_{Air}$ and $G_{Air}$ are the resistance and the conductance in purified air, respectively. For p-type material, the two formulas are swapped. 

As specified in the Introduction section, the chemical sensing performances of the fabricated array of sensors were evaluated towards four different chemical compounds that are among the most used food preservers: nitrogen dioxide (NO_2_), ethanol (CH_3_CH_2_OH), acetone ((CH_3_)_2_CO), and ozone (O_3_). This allows one to highlight the sensing performance and the differences between the materials in the screening of some compounds commonly found in the food industry, demonstrating the capability of the low power array to discriminate among different food preservers. Test gases with a certified composition supplied by SIAD SpA, (Bergamo, Italy) were mixed in a carrier of dry synthetic air by MKS Instrument mass flow controllers. A UV lamp was used to generate ozone close to the test chamber with a maximum concentration of 700 ppb. Ozone was then mixed with synthetic air to select the required gas concentration. The total flow inside the chamber was set at 200 SCCM. Short-term reproducibility was taken into account and evaluated for all chemical species and materials. Consecutive measurements were performed at the same concentration and on the same sensor. Results confirmed the very good repeatability of the measure, leading to an error less than 10% over four gas injections. All test compound concentrations were selected at much lower values than safety standards to stress the sensing performances of the portable array.

## 3. Results and Discussion

### 3.1. Morphological and Structural Characterization of Nanowires

**Figure 3:**
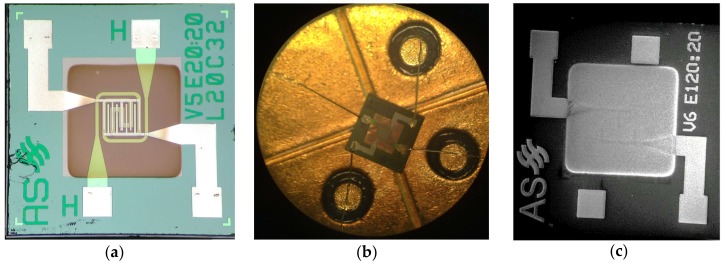
Optical images of WO_3_ device as-fabricated (**a**) and mounted with gold wires on TO package (**b**). Electrodes and heating elements are visible through the thin membrane. (**c**) SEM image of SnO_2_ device at low magnification (100×).

Prior to the wire bonding of the micro-hotplates on TO packages, devices were characterized morphologically by optical and FE-SEM microscopy to confirm the presence of the nanowires as well as their shapes and aspect ratios. Figure 3a,b report two optical images of a WO_3_ device. Due to the thermal oxidation technique used, nanowires could be patterned easily by simple shadow masking, and they were visible on top of the membrane and on the interdigited electrodes (dark area). Instead, it was not possible to precisely control the synthesis of SnO_2_ nanowires on the μHPs (Figure 3c), as the VLS mechanism was a random condensation process that could not be confined properly. The Pt catalyst promoted the growth of the nanowires in some specific regions, but, due to the presence of noble metals on the soldering pads, a minor growth happened on the pads as well. Most of the nanowires were located on the membrane, but this secondary growth on the soldering pads could have led to some issues during the soldering process. 

**Figure 4:**
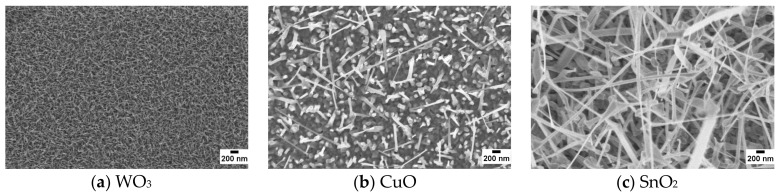
SEM pictures of WO_3_ (**a**), CuO (**b**), and SnO_2_ (**c**) nanowires directly synthesized on ams hotplates.

Figure 4 reports some SEM images of WO_3_, CuO, and SnO_2_ nanowires at the same magnification level. The density and the dimensions of nanowires are strongly dependent on the material and on the synthesis technique used. Tungsten oxide nanowires are very small and dense with an average diameter of about 20–30 nm [62]. The mat of copper oxide nanowires is less dense than WO_3_, and the average diameter is in the range of 70–100 nm [63]. In the case of tin oxide, a dense mat is obtained, but the nanowires are irregular and very long (few μm). It is more difficult to control the geometry of the wires, and thus their diameters are spread over a wide range (100–250 nm).

**Figure 5:**
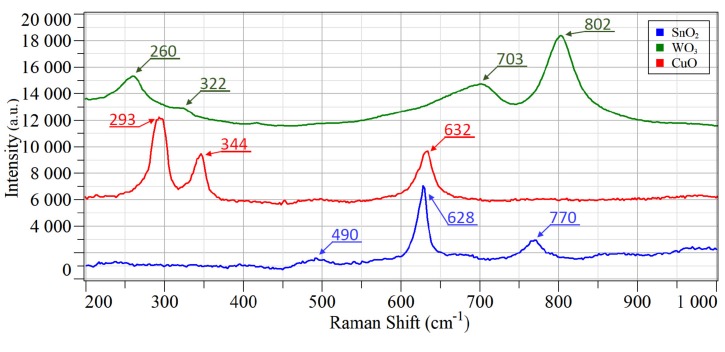
Raman spectra of SnO_2_, WO_3_, and CuO nanowires deposited on μHPs.

Raman spectroscopy is a non-destructive investigation technique that was already used for the characterization of the thermal properties of μHPs [73]. In this specific case, this powerful technique allowed for determination of the crystalline structure of the sensing material at micrometer size and directly synthesized on top of the hotplate. Indeed, thanks to the focalized laser source, it was possible to excite only the small area of the metal oxide, confirming that the synthesized nanostructures on the μHPs were crystalline for real. Raman spectra collected from the three different batches of samples are shown in Figure 5.

As reported previously, on tungsten oxide samples, the Raman shifts at 260, 322, 703, and 802 cm^−1^ could be attributed mainly to the monoclinic phase of WO_3_ (Figure 5, green line) [62,74,75]. The bending vibration of the O–W–O bonds generated the first two shifts. The peaks at 703 and 802 cm^−1^ instead corresponded to W–O–W stretching vibrations of the bridging oxygen. Raman measurements performed on CuO samples confirmed the monoclinic crystalline phase of CuO, commonly found in tenorite rocks (Figure 5, red line). The three predicted Raman active lattice modes were detected at 293 cm^−1^ (A_g_), 344 cm^−1^ (B_g_), and 632cm^−1^ (B_g_), in line with literature values [76]. Additionally, for the last observed batch of samples, Raman investigations identified shifts that matched the one reported in literature for the tetragonal phase of SnO_2_ (Cassiterite, Figure 5, blue line) [77]. The three detectable Raman active modes of tin oxide that were observed were E_g_ (490 cm^−1^), A_1g_ (628 cm^−1^), and B_2g_ (770 cm^−1^). 

### 3.2. Chemical Sensing Performances

**Figure 6:**
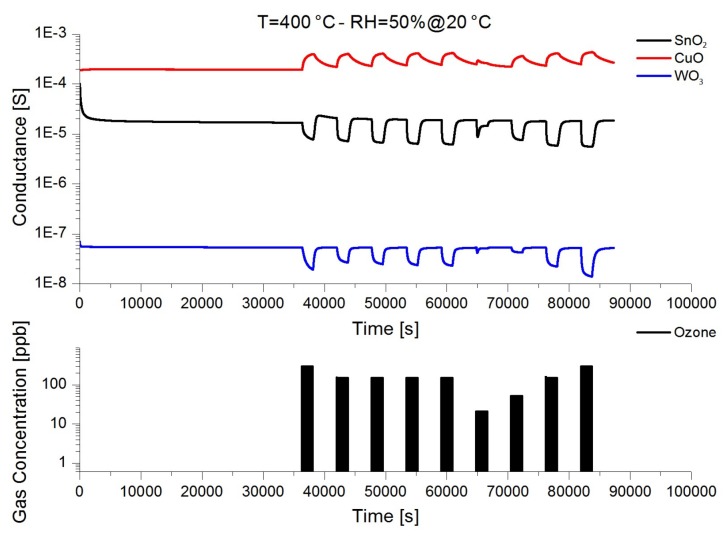
Dynamic response of SnO_2_, CuO, and WO_3_ nanowires in the presence of different concentration of ozone (300, 150, 150, 150, 150, 20, 50, 150, 300 ppb, respectively). Operating temperature for all sensing devices was 400 °C. Relative humidity was 50% at 20 °C.

As discussed already, the most common working principle of devices integrated in electronic noses is the change of the electrical conductance due to the interaction of the chemical species in the atmosphere with the surface of the sensing material [78]. Essentially, conductometric chemical sensors work as resistors in which the electrical resistance of the sensing layer is modulated by the adsorption or the interaction of chemical species with the surface. Figure 6 shows the electrical conductance of three different devices in the presence of ozone, a typical oxidizing gas. The effect of adsorbed species on the surface of the metal oxide depends on the nature of the semiconducting material. In the case of n-type materials such as SnO_2_ and WO_3_, the presence of a oxidizing gas, such as ozone, led to a reduction of free electrical charge on surface, thus decreasing the overall electrical conductance. In a p-type material such as CuO, the presence of oxidizing gas led to an increase of the conductance [79].

From the dynamic measure, it is possible to observe the excellent stability of the baseline of all three different devices at constant power mode, even during long-time operation. This is a fundamental requirement for the integration of reliable sensors into electronic noses; frequent sensors calibration is undesired because it requires personnel attendance, cannot be automated, and forces the instrument to stop [39]. Figure 6 also shows an example of the very good reproducibility of the measure. The response profiles of four consecutive ozone injections were almost identical in all samples, exhibiting an error less than 10% in short term investigations. Moreover, all devices completely recovered the baseline with no sign of surface contaminations or damage, even in the presence of a reactive gas such as ozone, which is known to be a very strong oxidizer.

In electronic noses, each sensor of the array can be powered at the desired temperature according to the specific compound fingerprints to detect. According to micro hotplate specifications, investigations were limited to 400 °C to avoid any damage during long-time operation, such as membrane breakdown caused by thermal stress. In this specific case, a temperature of 400 °C was selected as the optimal one for the detection of ethanol, acetone, and ozone, while for nitrogen dioxide, 200 °C was chosen. These values are in line with previous investigations [62,63,68]. 

**Figure 7:**
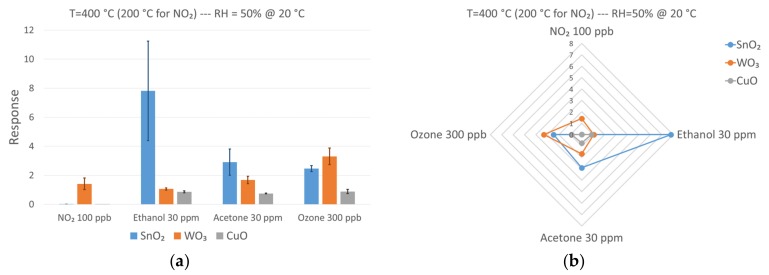
(**a**) Comparison of different sensing materials towards specific concentrations of target chemical compounds. (**b**) Polar graph of the response spectra of the devices. Operating temperature for all sensing devices was 400 °C towards ethanol, acetone, and ozone, while it was 200 °C for nitrogen dioxide. Relative humidity was 50% at 20 °C.

Each metal oxide deposited on the μHPs had different physical and electrical properties related to the surface chemistry and the defects in the crystalline structure [80]. Therefore, different sensor responses in the presence of specific concentrations of the target compounds were expected. To understand which was the most sensitive material towards each compound, the performances of the devices were compared directly at the same concentrations, as reported in Figure 7. The interest is to measure very low NO_2_ concentrations, which are quite difficult to detect with these kind of devices [81]. Among the three metal oxide devices investigated, only WO_3_ was able to produce an appreciable response towards 100 ppb of NO_2_, a concentration much lower than the safety standard requirements. This result was not unexpected; tungsten trioxide is well known in literature to have good sensing performance in detecting nitrogen dioxide [82]. Tin and copper oxide responses, however, were negligible. The asymmetry of the response between different materials is the key feature of electronic noses, because it helps patter recognition methods in the discrimination between different compounds. 

In these tests, tin oxide was mostly suited to detect volatile organic compounds (VOCs) such as ethanol and acetone. Interestingly, SnO_2_ was more sensitive to ethanol than acetone at the same concentration of 30 ppm. On the contrary, WO_3_ was more sensitive to acetone, even if its overall response was lower than SnO_2_. CuO was not able to compete with the other two materials in terms of response, but it did not seem to prefer either ethanol or acetone. Again, the different behavior of the metal oxides in VOCs sensing allowed a more efficient discrimination. Regarding ozone, tungsten trioxide was the most sensitive material toward a concentration of 300 ppb, followed by tin oxide. On the other hand, copper oxide response was significantly lower.

**Figure 8:**
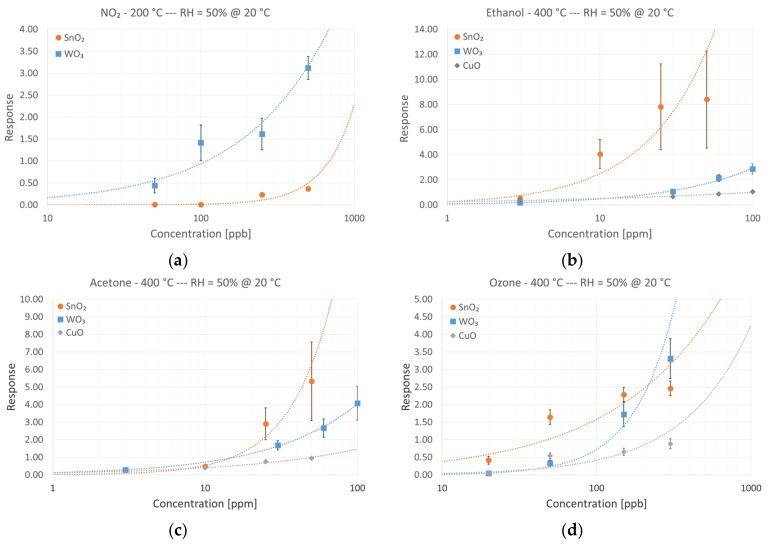
Calibration curves in the presence of different chemical species: (**a**) NO_2_, (**b**) ethanol, (**c**) acetone, and (**d**) ozone. Relative humidity was 50% at 20 °C.

In Figure 8, the responses of each different sensing material in the presence of various concentrations of target compounds are reported. Dashed lines in Figure 8 refer to a power-law fitting of data points according to the typical formula:

Response = A[gas concentration]^B^, (3)

where A and B are constants depending on the material and the target chemical species. Each data point is the average response of at least three different sensors from the same batch, nominally identical. Error bars refer to intrinsic measured variability between devices. 

Calibration curves confirmed the better performances of WO_3_ in detecting NO_2_, outperforming both tin oxide and copper oxide, which was not even reported due to very low response. Defining the detection limit of the devices as the gas concentration producing a unitary response (Response = 1), ≈100 ppb was identified as the limit for nitrogen dioxide.

Moving to VOCs, calibration curves confirmed the higher responses of SnO_2_ devices, especially towards ethanol. Detection limits (previously defined) were estimated as ≈5 ppm for ethanol and ≈15 ppm for acetone. However, an increased variability in the response of different tin oxide device was reported. This could be related to the synthesis process; it is more difficult to control the morphology and the dimensions of the nanowires, and this was reflected in sensor response. Tungsten and copper oxide devices performed worse, but responses from different devices of the same batch were more consistent. 

Ozone sensing showed mixed results. For high ozone concentration (>200 ppb), tungsten oxide devices exhibited better performance compared to tin and copper oxides. At moderate and low ozone concentrations (<200 ppb), tin oxide outperformed tungsten oxide devices. Copper oxide response was always less than the other two types of sensing devices. The detection limit for tin oxide devices was estimated at ≈40 ppb. Detection limits of the three different materials toward specific target compounds are reported in Table 1.

**Figure 9:**
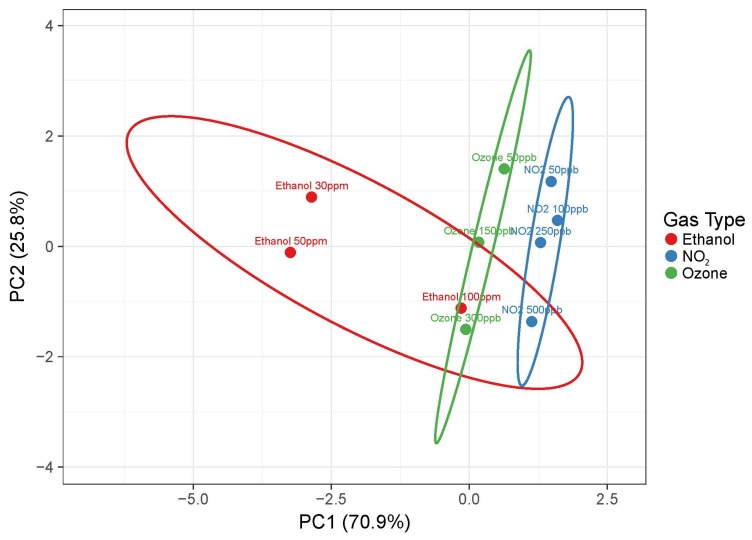
Pareto scaling was applied to rows; singular value decomposition (SVD) with imputation was used to calculate principal components. X and Y axis show principal component 1 and principal component 2 that explained 70.9% and 25.8% of the total variance, respectively. Prediction ellipses are such that with probability 0.95, a new observation from the same group falls inside the ellipse. N = 10 data points.

Principal component analysis (PCA) is by far the most used unsupervised data algorithm to manage the information coming from an electronic nose. It mainly consists of a linear extraction technique that reduces data dimensionality with a minimum loss of information, projecting them into lower dimensions (usually two or three) [20]. Figure 9 reports PCA performed using the data previously presented. As expected, efficient discrimination between ethanol and acetone was very difficult using only the three sensors integrated in the array. Therefore, acetone was not included in this representation. However, it would be possible to add new sensors to the array when integrated in the electronic noses, potentially increasing the number of different sensors up to thousands, which would allow for more likely discrimination of specific fingerprints [78]. Nevertheless, WO_3_, SnO_2_, and CuO devices were able to discriminate between ethanol, ozone, and nitrogen dioxide at low concentrations, demonstrating the capability of the fabricated sensor array to distinguish the correct food preserver.

## 4. Conclusions

Electronic olfaction systems are starting to be used largely in food safety and security fields due to high reliability, low cost, easy use, and the possibility of online monitoring. While research is focusing on the enhancements of their performances to promote further use of such systems in the food industry, a special attention is devoted to reducing the power consumption of each single sensor to allow the fabrication of portable or battery operated equipment. 

In this work, a simple sensor array based on copper, tin, and tungsten oxide nanowires was prepared, and its performance in the detection of common food preservatives was evaluated. The nanowires in the sensing array were synthesized on commercial low power micro-hotplates from ams Sensor Solutions Germany GmbH (Reutlingen, Germany). This novel sensor array combines the advantages of conductometric metal oxides together with the increased sensing performances of nanowire technology and the reduced power consumption from silicon MEMS technology. The three sensing materials were characterized to investigate the morphology and the structure of synthesized nanowires by using FE-SEM and Raman spectroscopy. 

The fabricated array was tested towards four different food preservatives commonly used in the food industry—nitrogen dioxide, ethanol, acetone, and ozone. Tungsten oxide resulted as the most sensing material to detect nitrogen dioxide, even in low concentrations (100 ppb). Tin oxide, however, showed higher performance in detecting VOCs such as ethanol and acetone. The response of copper oxide devices was always less than the other two materials but was still measurable. 

Results confirmed the ability of the sensing array to detect concentrations of food preservers much smaller than safety standard requirements. The sensing performances of these different materials appeared to be complementary; the combination of all sensor readouts may provide significant information when integrated in arrays or e-noses. Future research activities aim at evaluating the performance of this novel e-nose in real applications, such as the real-time monitoring of specific food products for quality and safety analysis. Moreover, the use of a common technological platform allows the mass production of these sensors, helping to reduce the fabrication costs. Finally, the very small thermal inertia of the micro hotplates allows fancy operating modes, such as discontinuous operation or pulsed temperature [41]. This is considered the frontier of sampling, because it not only reduces the power consumption of the system but also enables the use of more advanced data algorithms, such as advanced fuzzy and pattern recognition, which can give a boost in the accuracy of the fingerprint and the aroma recognition [83]. 

## Figures and Tables

**Table 1:** Detection limits of the three different materials toward target chemical compounds.

	NO_2_	Ethanol	Acetone	Ozone
SnO2	>1 ppm	5 ppm	15 ppm	40 ppb
WO3	100 ppb	25 ppm	15 ppm	150 ppb
CuO	>1 ppm	40 ppm	50 ppm	300 ppb
